# From product to knowledge: the life cycle of health technologies 2.0 as a framework for health system improvement

**DOI:** 10.3389/fphar.2026.1869110

**Published:** 2026-08-10

**Authors:** M. Otte, I. Gutiérrez-Ibarluzea, H. P. Dauben

**Affiliations:** 1 Innovationsinstitut für Gesundheit und Gesellschaft gUG (I2GG gUG), Jüchen, Germany; 2 EuroScan international network e. V., Cologne, Germany

**Keywords:** evidence-based medicine, health system governance, health technology assessment, knowledge generation, life cycle framework, real-world evidence, stakeholder engagement

## Abstract

**Background:**

Existing life cycle frameworks for health technologies have mainly adopted a product-oriented perspective. They treating evidence as a linear input to discrete regulatory and coverage decisions, and failing to capture the iterative, context-dependent, stakeholder-specific nature of knowledge generation needed for contemporary health-technology governance.

**Objectives:**

This article introduces the Life Cycle of Health Technologies 2.0 (LC 2.0), an updated conceptual framework building on the Life Cycle of Health Technologies (2017, LC 1.0). It traces the evolution from LC 1.0 to LC 2.0, conceptualises LC 2.0 as a knowledge life cycle, and outlines illustrative use cases. LC 2.0 integrates existing conceptual approaches to explore how knowledge about a health technology develops across stakeholder groups across different processes. In line with the Health for All Policies concept, it links this knowledge life cycle to societal, system and patient needs within and beyond the health sector.

**Methods:**

A mixed-methods approach was applied comprising multi-stakeholder workshops and use-case validation across four technology types.

**Results:**

Workshops identified revision requirements in LC 1.0 across macro, meso and micro levels, centring on structural inflexibility, implicit post-market learning and limited integration of contextual and stakeholder knowledge. The LC 2.0 framework addresses these through an infinity-loop structure with six phases and twelve subprocesses. It makes knowledge generation an explicit function at every phase and maps knowledge requirements to stakeholder roles. It also incorporates flexible, non-sequential pathways, including a cross-phase transition point (X) as governance decision point between research and development and between continued optimisation and more fundamental revision.

**Conclusion:**

LC 2.0 reconceives the health-technology life cycle as an iterative knowledge-generation and decision-supporting service, structuring what must be learned, by whom and when, to support appropriate technology use, needs-driven innovation and regulator-appropriate evidence generation, and to provide a shared reference framework for stakeholders inside and adjacent to healthcare and regulatory systems. This shared structure also facilitates easier exchange between different health-system contexts.

## 1 Introduction

Health technologies, encompassing medicines, medical devices, diagnostics, digital applications, procedures, public health programmes and organisational systems, are developed with the primary objective of preventing, diagnosing, treating or improving health and healthcare delivery (EuroScan international network, 2025). Beyond their intended clinical effects, health technologies are expected to contribute to improvements in quality of life, system performance and population health ((Banta and Thacker, 1990), (Jonsson et al., 2002)). Their implications extend further, reaching organisational, economic and societal dimensions, including long-term effectiveness and sustainability considerations (Greer et al., 2023; Mayer-Foulkes et al., 2021).

Health technologies are best understood as sociotechnical phenomena. Their introduction and use reshape both technical components — tools, infrastructures and procedures — and social components, including professional roles, organisational routines, institutional norms and user practices (Greenhalgh et al., 2017; Li, 2010; Papoutsi et al., 2021). Effects emerge from interactions between technologies, actors, and contexts including cultural aspects, rather than from technological properties in isolation (Bergström et al., 2020). Understanding and governing these interactions, including associated questions of ethics and social fairness, has long been a central concern of health policy (Barbazza et al., 2015; Bellemare et al., 2018; Culyer and Bombard, 2012).

Given this complexity, life cycle frameworks have been employed to structure understanding, support comparison and facilitate decision-making under uncertainty (Regier et al., 2022; National Academies of Sciences Engineering, and Medicine et al., 2017). Such frameworks do not aim to reproduce reality in its entirety. Instead, they provide simplified representations that highlight selected elements and relationships, offer orientation, enable communication across disciplines and decision levels and make implicit assumptions explicit (Johnson and Busemeyer, 2010). In healthcare, life cycle frameworks have been used to organise evidence generation, inform planning and support coordination between research, development, assessment, use and disuse (Wilson et al., 2014).

Despite their widespread use, existing life cycle frameworks only partially capture the processes through which health technologies are developed, introduced, implemented, and sustained within complex healthcare systems (Nason, 2023; Tarricone et al., 2022; Sittig et al., 2023). Empirical evidence indicates that many technologies encounter barriers to uptake or fail to deliver anticipated benefits. Often this is not because of insufficient technical performance, but due to macro-level conditions such as regulatory requirements, funding arrangements, governance structures, or market access constraints (Sareban et al., 2025; Iqbal and Biller-Andorno, 2022). Many frameworks treat these conditions are treated as external background factors rather than being systematically integrated into the core framework logic, limiting their explanatory power and practical utility (Nilsen and Bernhardsson, 2019).

Even when regulatory and funding requirements are met, challenges frequently arise during implementation when technologies are not aligned with organisational contexts. Technologies may fail to integrate into existing care pathways, disrupt established workflows, require unavailable resources, or conflict with professional roles and routines (Safi et al., 2018). Organisational conditions are addressed implicitly or only at specific points within existing frameworks (Moullin et al., 2015). At the individual level, the use of health technologies depends on the awareness of health technologies and engagement of patients, healthcare professionals and other end users, whose perspectives and experiential knowledge remain underrepresented in life cycle frameworks (West et al., 2025; European Commission, 2026a).

The Life Cycle of Health Technologies framework introduced by Gutiérrez-Ibarluzea et al. (LC1.0) represented a significant conceptual advance by framing health technologies not only as products, but also as part of broader sociotechnical knowledge processes, understood as the ways in which information, experience and evidence are generated, shared and used through interactions between technologies, organisations and social actors (Gutierrez-Ibarluzea et al., 2017). In the article, knowledge is defined in a broad, operational sense that goes beyond formal evidence from study hierarchies. It encompasses not only research evidence, but also experiential insights, contextual information and value-based judgements that are generated and exchanged between stakeholders when governing health technologies. The framework highlights that innovation emerges from interactions between technological characteristics and the contexts in which technologies are developed, assessed, used and disused—encompassing regulatory frameworks, organisational arrangements, social practices, and values considerations (Oortwijn and Sampietro-Colom, 2022; Otte et al., 2024).

Since the publication of the original framework, the landscape has evolved and become more interdisciplinary. Emerging technology groups such as digital health applications, Advanced Therapy Medicinal Products (ATMPs) and artificial intelligence-based tools have shortened development cycles, altered risk–benefit profiles. They also created new demands for validation, including non-clinical outcomes that focus on patients and societies. In indications where no or only suboptimal interventions are available, decisions increasingly concern how much uncertainty and risk health systems are willing to accept, rather than technical feasibility. This makes it essential to define explicit system-level boundaries to avoid unrealistic promises and unsustainable spending. To anticipate possible issues along the development process, health systems are now more actively involved in innovation at earlier phases through instruments such as early dialogues, early advice processes, regulatory sandboxes, pre-commercial procurement and scientific advice by public agencies and organisations (Ibargoyen-Roteta et al., 2022; Hofer et al., 2015; Gutierrez-Ibarluzea et al., 2022; BCIHI, 2026). Patients and other stakeholders are increasingly engaged through patient-reported outcome and experience measures to improve value assessment approaches beyond economic evaluations. Health Technology Assessment (HTA) has evolved towards more dynamic approaches including reassessment over time, and societal expectations have expanded to include equity, sustainability, and social participation (Imeshtari et al., 2025).

Against this background, there is a need for a framework that goes beyond describing what happens to a health technology across its product life cycle. It should explicitly address what is learned, by whom, and when and how that learning drives system improvement. Helping to reduce uncertainty, increase appropriate use, and efficient research and development. The present study explores the Life Cycle of Health Technologies 2.0 (LC 2.0) as an approach to integrate technical, product-oriented descriptions and evidence into a service-oriented knowledge perspective within a shared reference structure for societies. Three main research questions guide the study:

- Q1: What additional requirements emerge from recent developments compared to the original Life Cycle of Health Technologies (LC 1.0)?
- Q2: What types of knowledge are required by different stakeholder groups to support decision-making and acceptance across health technology life cycle?
- Q3: How can knowledge hierarchies be interpreted and adapted in a phase-specific manner within life cycle contexts?

## 2 Methodology

This work employed a mixed-methods approach combining participatory workshops, use case validation, narrative literature synthesis and iterative framework development (Green et al., 2015; Author Anonymous, 2024). The methodological design progressed sequentially. Stakeholder workshops examined LC 1.0 and generated a draft framework addressing Q1. Use case testing validated the draft across diverse technology types. A narrative literature synthesis characterised stakeholder evidence needs for Q2, and conceptual analysis developed phase-specific evidence frameworks for Q3. A final integration step consolidated all findings into the LC 2.0 framework.

### 2.1 Stakeholder workshops

To identify requirements for revising the LC1.0, a series of stakeholder workshops was conducted between November 2022 and December 2024. Workshops were organised across multiple projects and formats.

In all workshops, the LC 1.0 framework was used as a shared reference structure at the outset to establish a harmonised understanding of the overarching life-cycle logic among participants. After a brief introduction to the framework, each session focused on the specific thematic agenda, with discussions repeatedly relating experiences and challenges back to the life-cycle structure. This design supported that comments on gaps, misalignments or missing elements could be directly situated in terms of phases, transitions and decision points.

Originally developed from a health technology assessment perspective, LC 1.0 was used in these workshops as a tool to identify where different stakeholder groups struggled to make sense of the technology life cycle and how their perspectives could be brought onto a shared footing. Plenary discussions and group work outputs were documented through facilitator notes and workshop minutes. Flip charts and whiteboards were captured digitally.

Participants were sampled to represent eight stakeholder groups: regulatory authorities, HTA agencies, healthcare professionals, patient organisations, industry representatives, healthcare payers, investors, representatives from other policies, and researchers/academia. This composition supported coverage of macro-level regulatory and policy perspectives, meso-level organisational considerations and micro-level technology end-users and patient viewpoints (Carter et al., 2025). Following each workshop, the documentation was reviewed to extract change requests to the framework, which were collated into thematic clusters of revision requirements.

### 2.2 Use case validation

To assess applicability and robustness, the draft framework was tested against four purposively selected technology categories (Pharmaceuticals, digital health technologies, public health screening programmes and ATMPs). Pharmaceuticals refers to small molecules and biologics, whereas ATMPs denote gene, cell and tissue-engineered products governed by the dedicated EU ATMP regulation and centralised EMA procedures (European Medicines Agency, 2026a). These categories were selected to span well-established regulatory pathways (pharmaceuticals), rapidly evolving software-based technologies (digital health), population-level interventions with complex implementation (screening programmes) and specific EU regulatory pathways for ATMPs, which typically rely on small trial populations and extended post-authorisation evidence generation (European Medicines Agency, 2026b; European Medicines Agency, 2026c; Irinyi et al., 2026).

For each category, an exemplar use case was developed, based either on real-world cases (biosimilar adoption in low- and middle-income settings, paediatric familial hypercholesterolaemia screening) or on composite scenarios that combined recurring patterns from the literature and stakeholder workshops (digital decision-support tools in antenatal care, outcome-based contracts for ATMPs). A detailed overview of these use cases is provided in Supplementary Table S1. Each use case was mapped onto the phase structure of the draft life-cycle framework to document represented phases and subprocesses, skipped or merged phases and additional activities or evidence needs, and these mappings informed iterative refinement of phase transitions, feedback loops and the role of real-world evidence.

### 2.3 Literature synthesis on stakeholder knowledge requirements

A narrative literature synthesis was conducted to characterise the types of knowledge required by different stakeholder groups across the life cycle to support decision processes. Due to the heterogeneity of stakeholder roles, we drew on different source types for each group.

For healthcare professionals, searches were conducted in PubMed, Scopus and EMBASE using combinations of terms related to health technologies, life-cycle phases, evidence requirements and stakeholder perspectives. Conceptual and empirical publications were included that discussed evidence requirements across different stakeholders or life-cycle phases and excluded technical clinical-trial reports that did not address decision-making or governance.

For patients and patient organisations, additional searches targeted grey literature, reports from patient advocacy groups and guidance on patient-reported outcomes and experiences. For payers, HTA agencies and regulatory authorities, we primarily reviewed policy documents and methodological guidance from national and international HTA organisations, regulatory agencies and WHO, complemented by relevant academic publications. For industry, investors and cross-sectoral policy representatives, we considered reports, position papers and methodological documents issued by professional associations, industry bodies and public-sector organisations.

A detailed description of the search strategy is provided in Supplementary Table S2, following the recommendations by Haas et al. (2013). Identified knowledge requirements were extracted, mapped to the stakeholder groups and synthesised into a stakeholder–knowledge requirement matrix.

### 2.4 Phase-specific evidence framework

The 2011 Oxford Centre for Evidence-Based Medicine (OCEBM) Levels of Evidence framework was applied as an interpretive tool. Unlike the earlier, more linear Oxford evidence hierarchies that are often summarised as a single pyramid, the 2011 OCEBM framework organises evidence by clinical question type and emphasises the “best available” design for a given question rather than a universal ranking of study types. It explicitly distinguishes between diagnosis, prognosis, treatment benefit, treatment harm and screening questions and allows different study designs to occupy the highest level depending on the decision context (Centre for Evidence Based Medicine, 2025). This question-based logic was applied to examine how evidence and quality standards should be interpreted across different life cycle phases, from early development to post-market surveillance to reassessment and beyond.

### 2.5 Framework consolidation and final testing

The validated draft conceptual framework, stakeholder evidence mappings and phase-specific evidence framework were integrated through a systematic synthesis process (Child and Shaw, 2023). Multi-level contextual factors were embedded across all phases and stakeholder engagement points were refined to reflect phase-specific evidence needs and decision roles (Boyer et al., 2018). The integrated LC 2.0 framework was then presented to workshop participants for structured feedback on comprehensibility, accuracy, practical utility and conceptual coherence. Revisions were applied iteratively to preserve internal consistency and structural integrity.

## 3 Results

### 3.1 Additional requirements compared to LC 1.0 (Q1)

Stakeholder workshops revealed revision requests in LC 1.0 across three system levels.

At the macro level, the framework was perceived as sequential and inflexible. Participants highlighted that skipping, compressing or parallelising phases is common in practice, but not explicitly represented. They also noted that technologies frequently follow partial life cycles, enter at different points of evidence to decision generation or exit without completing all phases. Important macro-level dynamics including adaptive regulatory pathways, early health system engagement through instruments such as early dialogue, regulatory sandboxes and pre-commercial procurement, and alignment with normative goal systems such as Health-for-All-Policies and the Sustainable Development Goals were absent.

At the meso level, an observation made was the absence of a distinction between three distinct levels of abstraction: the product, the technology, and the technology group. These concepts are not interchangeable.

- A product is a commercially and regulatorily defined artefact that is identifiable, certifiable and subject to market authorisation. It can be regulated under frameworks such as the EU Medical Device Regulation, assigned a specific CE identification and evaluated for safety and efficacy under controlled conditions; the entity sold by a company is a product (for example, a specific MRI scanner of class IIb, regulatable and testable as such).
- A technology, by contrast, is an abstract application or procedure that subsumes similar products; MRI imaging is a technology that encompasses multiple MRI scanner products and associated protocols. This aligns with HTA definitions that describe a health technology as an intervention or method (test, device, medicine, procedure, programme or system) rather than a single marketed item.
- A technology group is a thematic cluster of related technologies; imaging procedures (for example, MRI, CT, ultrasound and X-ray modalities) constitute a technology group that can be assessed at a more aggregate level in health-system and policy analyses. Such groupings are commonly used in taxonomies of medical devices and interventions developed for HTA and regulatory or reporting purposes.

Distinguishing explicitly between products, technologies and technology groups is therefore essential, because regulatory processes typically operate at the product level, many research and evaluation activities address the technology level, and strategic planning or disinvestment decisions are often made at the technology-group level.

LC 1.0 did not draw these distinctions. As a result, participants emphasised that making these levels explicit would be necessary to adequately represent how technologies are embedded in healthcare pathways, service frameworks and broader service ecosystems. Systematic feedback from routine use into earlier phases was only partly addressed, particularly with the concept of ‘health needs assessment’.

At the micro level, participants highlighted the absence of systematic feedback from individual users and patients into the life cycle. Patient co-design, user experience and values-based dimensions (societal values) were not represented. Practice-based learning and user-driven adaptation were not operationalised, despite their recognised importance for technology acceptance and sustained use.

Synthesising these findings, three principal dimensions of extension were identified:

- a temporal dimension requiring greater flexibility and non-linear phase representations;
- an actor dimension requiring explicit representation of a broader range of stakeholders with distinct roles and decision contexts;
- a knowledge dimension requiring systematic integration of evidence beyond clinical efficacy and safety, including implementation outcomes, organisational readiness, user experience, equity impacts, sustainability considerations, and societal value creation.

### 3.2 Stakeholder-specific evidence requirements (Q2)

The narrative synthesis identified evidence requirements across the mentioned stakeholder groups. Evidence needs vary by primary decision context, the type of judgement to be made and the timing within the life cycle.

Regulatory authorities primarily require evidence to support marketing authorisation and post-market safety oversight. Pre-market decisions rely on data on quality, safety and efficacy, typically generated through clinical trials that are designed to maximise internal validity and support causal conclusions about benefit–risk profiles, and are supported by manufacturing and risk-management documentation. After market entry, technology-vigilance data and real-world evidence become increasingly important for ongoing benefit–risk assessment and labelling decisions. Real-world evidence (RWE) denote clinical and health system evidence derived from analysis of data collected outside of prospectively controlled trial settings. This includes registries, electronic health records, claims data, pragmatic trials and routine patient-reported outcomes (Administration FaD, 2026; Dang, 2023). Because randomised controlled trials alone may not capture the full spectrum of adverse events or long-term and rare outcomes, RWE in this phase functions as a bridge between routine outcome observation and strengthening or challenging causal inferences about the technology’s effects in broader practice (Barbier et al., 2025).

HTA agencies focus on reimbursement, coverage and resource allocation and deallocation decision support. At market entry, they require clinical effectiveness evidence that is sufficiently robust to support causal conclusions about comparative benefit and harm, typically based on well-designed randomised or otherwise controlled studies, combined with economic evaluations, including cost-effectiveness and budget impact analyses. Post-adoption, reassessment decisions depend on real-world effectiveness, implementation outcomes (e. g. reach, acceptability, feasibility and fidelity of use in routine care), societal impacts such as equity and fairness and patient preferences (Wieseler et al., 2026).

Healthcare professionals require clinical effectiveness and safety data in relevant populations, as well as practical information on usability and workflow integration. During routine use, locally generated audit data and patient-reported outcomes complement trial evidence. Long-term safety and effectiveness data support post-adoption decisions (Rivera et al., 2019).

Patients and patient organisations emphasise understandable information on benefits and harms, quality of life impacts, and treatment burden. Patient-reported outcomes and experience data from real-world use are particularly relevant, as is evidence on accessibility and equity of access (Brogan et al., 2017).

Industry and technology developers require evidence across multiple phases: from needs assessments and proof-of-concept data in early development, to safety, efficacy, and economic evidence for regulatory and reimbursement submissions, to post-market real-world evidence for product life-cycle management and expanded indications, that could involve rare adverse events or effects not identified in selected populations (Kardas, 2022).

Healthcare payers and commissioners require cost-effectiveness and budget impact evidence at adoption, utilisation patterns and organisational impacts during implementation, and real-world performance data for reassessment and outcomes-based arrangements, including adverse events (Ádám et al., 2022).

Investors, encompassing both private and public investors, require evidence to inform funding and procurement decisions across different phases of the life cycle and risk/benefits balances. Private investors, including venture capital and strategic industry investors, focus primarily on early development phases, where proof-of-concept data, feasibility evidence and preliminary safety and efficacy signals inform decisions on whether to commit resources to further development. Demonstrable market potential, a credible regulatory pathway, and early health economic modelling are particularly relevant at this phase (Safavi et al., 2020).

Public investors, including research funders, public procurement agencies, and bodies engaged in pre-commercial procurement, operate with a different logic: their evidence needs are oriented toward societal return on investment, alignment with public health priorities, and the capacity of a technology to address articulated system needs. Instruments such as pre-commercial procurement explicitly require evidence that a technology responds to a defined public sector need and can be developed to meet specific performance criteria (European Commission, 2026b).

Representatives from other policy sectors, including social policy, education, environment, labour, and economic development, constitute a stakeholder group whose evidence needs are frequently overlooked in product-oriented life-cycle frameworks, yet are of growing importance as health technologies are increasingly assessed against cross-sectoral objectives. Under frameworks such as Health-for-All-Policies and the Sustainable Development Goals, health technologies are expected to contribute not only to clinical outcomes but also to broader societal goals, including equity, social participation, environmental sustainability, and economic productivity (St-Pierre, 2016; Assembly, 2015; Boitard et al., 2024). Policy representatives from these sectors require evidence on non-health impacts and co-benefits: effects on employment and workforce participation, environmental footprint of manufacturing and disposal, implications for social inclusion and accessibility, and contribution to SDG targets. The integration of these stakeholders into the life cycle framework reflects the understanding that the value of health technologies cannot be assessed in health system terms alone.

### 3.3 Phase-specific interpretation of evidence hierarchies (Q3)

Applying the 2011 Oxford Centre for Evidence-Based Medicine (OCEBM) Levels of Evidence framework as an interpretive tool demonstrated that evidence needs vary with decision context and life-cycle phase (Durieux et al., 2013). In early development phases, decision-making focuses on technical feasibility, proof of concept and preliminary safety. At this phase, laboratory studies, preclinical investigations, mechanistic research, small pilot studies, qualitative research and expert opinion can represent the best available evidence for the questions at hand, because they are able to address foundational uncertainties before comparative trials are ethically or practically justified. Within the 2011 OCEBM framework, these evidence forms may occupy lower levels of the traditional hierarchy, but their adequacy is judged by their capacity to reduce uncertainty sufficiently to justify progression rather than by a universal ranking of designs. Expecting high-level comparative evidence such as large randomised controlled trials in very early phases would not only inhibit innovation, but it would also often be ethically problematic, because exposing patients to experimental interventions without prior mechanistic and safety insight is not acceptable. This phase-specific reading of OCEBM is consistent with approaches such as the GRADE framework, in which decisions are based not only on the certainty of evidence but also on explicit trade-offs between benefits, harms, values and resource use along the evidence-to-decision pathway (Goldet and Howick, 2013).

During regulatory approval phases, the decision context shifts toward marketing authorisation, safety, efficacy, and benefit–risk assessment. Randomised controlled trials (RCTs) represent the reference standard for demonstrating efficacy, supported by systematic reviews and comprehensive safety data (European Medicines Agency, 1998). The OCEBM framework also recognises that for rare diseases or breakthrough technologies, alternative study designs — such as single-arm trials or historical controls — may be scientifically appropriate (Tenhunen et al., 2020). In the HTA and reimbursement phase, decisions address cost-effectiveness, budget impact and coverage. These decisions draw on comparative effectiveness evidence, health economic modelling, real-world data and patient preference studies (Mendell et al., 2023).

In implementation phases, organisational adoption, workflow integration, and capacity building become the focus. Evidence from implementation science, process evaluations, feasibility studies, qualitative research and local audit data carries high contextual validity, even if ranked lower in classical hierarchies (Hamilton and Finley, 2019; Rogers et al., 2021).

During post-market and real-world use phases, ongoing benefit–risk monitoring and reassessment depend on real-world data from registries, claims databases and electronic health records, alongside pragmatic trials and patient-reported outcomes. For reassessment, the totality of accumulated evidence may show a lack of effectiveness or safety concerns, comparative data demonstrating superior alternatives, and qualitative evidence on implementation failures, which together inform indication change, withdrawal or restriction (Pawson, 2019).

## 4 The life cycle of health technologies 2.0 conceptual framework

### 4.1 Structure and visual representation

**FIGURE 1:**
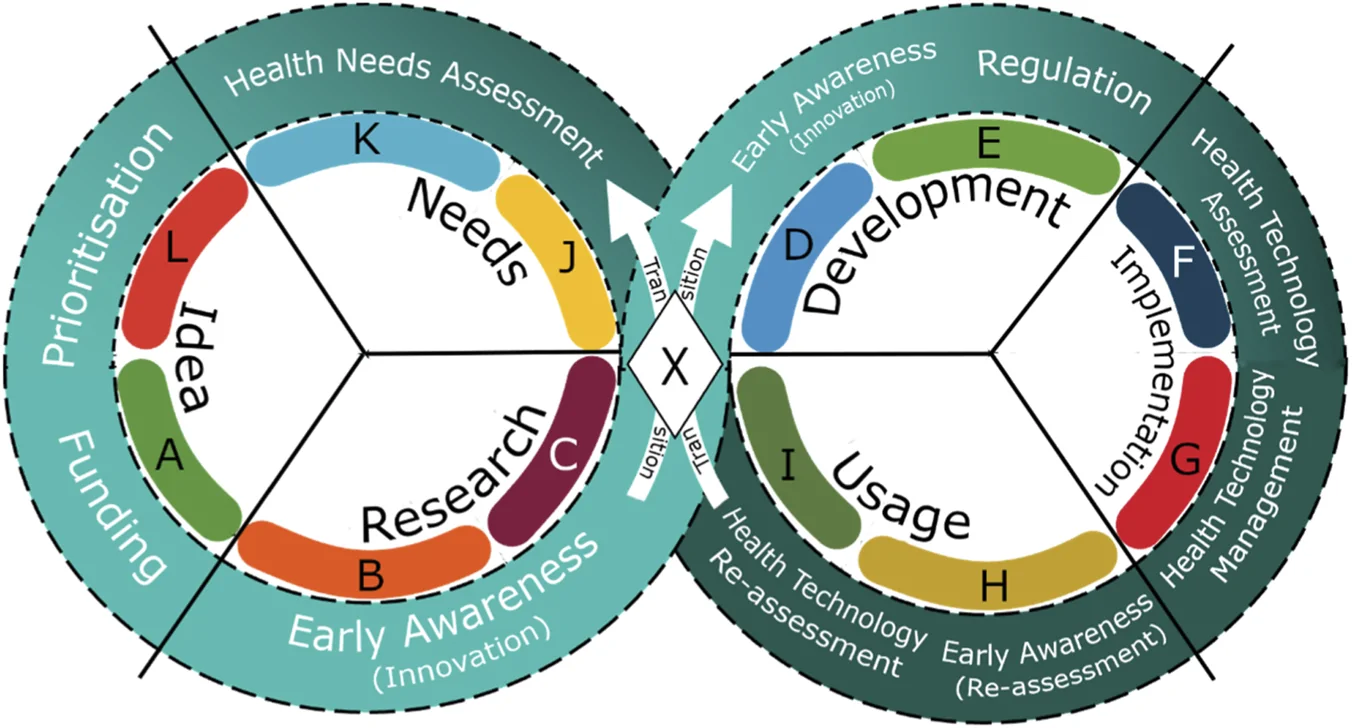
Life Cycle of Health Technologies (LC 2.0) for Knowledge. The LC 2.0 is represented as an infinity loop with six phases (Needs, Idea, Research, Development, Implementation, Usage) and twelve subprocesses **(A–L)**, indicating typical decision-oriented activities within each phase. Transition point X marks the main governance decision node, linking Research to Development and Usage back to earlier phases. Needs: J, Interpretation of reassessment; K, Translation of gaps. Idea: L, Normative prioritisation of needs; A, Translation of demands. Research: B, Operationalisation of research questions; C, Initial empirical evidence generation (e.g., pre-clinical, clinical, qualitative). Development: D, Translation of evidence into concrete technological and organisational solutions; E, Formal regulatory assessment. Implementation: F, Coverage and reimbursement; G, Adoption. Usage: H, Routine use; I, Reassessment. X Transition point X marks a governance decision node.

The LC 2.0 framework integrates the findings from all three research questions into a unified conceptual framework presented as an infinity loop structure (Figure 1). The visual representation builds on the foundational insight of LC 1.0 that health technologies evolve through interconnected product- and knowledge-related processes and extends it through the systematic integration of evidence-based life cycle instruments: Health Needs Assessment (HNA), Early Awareness and Alert systems (EAA), Health Impact Assessment (HIA), Health Technology Assessment (HTA), and Health Technology Management (HTM). Together, these instruments are embedded within the infinity loop to illustrate continuous feedback, non-linear progression, and learning across phases.

### 4.2 Six phases and their knowledge generation functions

The framework is structured around six phases positioned along the infinity loop. Each phase groups conceptually related processes and reflects a distinct but interconnected knowledge generation function.

The *Needs phase* marks an entry point of the life cycle and focuses on the articulation of prioritised health needs, referred to as demand, that warrant innovation and collective action. This phase introduces pull mechanisms alongside traditional push mechanisms of innovation. While push mechanisms originate from technological opportunities and scientific advances, pull mechanisms are driven by articulated system needs, service gaps, and policy objectives. Needs are analytically identified deficiencies derived from learning and reassessment; demands arise when these needs are prioritised and legitimised through societal and political decision-making. By anchoring innovation initiation in population health objectives, this phase shifts the starting point of the life cycle from predominantly technology-driven push dynamics toward governance-driven pull dynamics.

The *Idea phase* is the interpretive space in which gaps identified through reassessment are translated into innovation hypotheses, alternative solution pathways, and problem reformulations. By supporting learning, conceptual renewal, and innovation triggers, this phase establishes the return loop from real-world use back to new conceptualisation, operationalising the infinity loop as a genuine learning cycle rather than a circular repetition.

The *Research phase* translates prioritised demands and innovation concepts into research questions and study designs. It focuses on systematic evidence generation to assess feasibility and safety, encompassing preclinical and clinical investigations that test conceptual assumptions under controlled conditions.

The *Development phase* builds on evidence generated in Research and focuses on translating validated concepts into implementable technologies. It covers technology refinement, product development, and preparation for regulatory assessment, thereby supporting technical, regulatory, and organisational readiness for subsequent implementation.

The *Implementation phase* addresses decisions on adoption, coverage, and organisational integration. It encompasses HTA, coverage decisions, and implementation planning — translating developed and regulated technologies into routine use within healthcare organisations.

The *Usage phase* represents the routine application of health technologies in real-world care settings, focusing on post-market surveillance, real-world evidence generation, and health technology reassessment.

The Needs phase then marks the renewed entry point of the life cycle and focuses on the articulation of prioritised health needs, referred to as demand, that warrant innovation and collective action. This phase introduces pull mechanisms alongside traditional push mechanisms of innovation. While push mechanisms originate from technological opportunities and scientific advances, pull mechanisms are driven by articulated system needs, service gaps and policy objectives. Needs are analytically identified deficiencies derived from learning and reassessment; demands arise when these needs are prioritised and legitimised through societal and political decision-making.

### 4.3 Twelve subprocesses and decision-oriented activities

Within the six phases, the framework operationalises governance functions through twelve subprocesses (A–L). These subprocesses specify typical decision-oriented activities and outputs, without implying a mandatory or universal sequence. The illustrative sequence A to L clarifies how problem articulation, evidence generation, development, implementation, use, and learning can be linked. In practice, technologies may follow different routes, enter at different subprocesses, or move non-sequentially between them, and the LC 2.0 framework is explicitly designed to support this flexibility while still providing clearly defined subprocesses as orientation points for governance and evidence planning. The subprocess structure thus serves as a heuristic device to make governance functions and decision dependencies visible and comparable across technology types. Table 1 provides an overview of phases, subprocesses, and their core knowledge generation activities.

**TABLE 1:** Life cycle of health technologies 2.0: phases, subprocesses, and core knowledge activities.

Phase	Subprocess	Core knowledge activity	Illustrative output
Idea	A	Translation of demands into action-guiding problem definition	Decision-relevant problem definition
Research	B	Operationalisation into research questions and study protocols	Research questions and study protocols
Research	C	Initial empirical evidence generation (e.g., pre-clinical, clinical, qualitative)	Initial decision-relevant evidence
Development	D	Translation of evidence into technological and organisational solutions	Implementable solution concept
Development	E	Formal regulatory assessment or equivalent approval	Regulatory decision or conditional authorisation
Implementation	F	Coverage and reimbursement decisions	Adoption, restriction or rejection decision
Implementation	G	Organisational adoption, workflow redesign, capacity building	Operationally deployable intervention
Usage	H	Routine use; generation of utilisation, outcome and experience data	Real-world performance data
Usage	I	Reassessment of real-world performance against anticipated benefits	Identified gaps and performance signals (e.g., for disinvestment)
Needs	J	Synthesis and interpretation of reassessment findings	Structured gap clusters
Needs	K	Translation of gaps into descriptively formulated needs	Identified (non-prioritised) needs
Idea	L	Normative prioritisation and legitimisation of needs, funding priorities	Prioritised and legitimised demands, funding requirements

### 4.4 Transition point X and non-sequential pathways

A distinctive structural feature of LC 2.0 is the transition point (designated “X”) in the infinity loop. This point represents a cross-phase governance decision space connecting the two-halves of the loop and enabling fundamental redirection of the life cycle trajectory.

In forward progression (C to D), X functions as the decision point between Research and Development, where accumulated evidence is judged sufficient to justify translation into a concrete product or system-level solution; alternatively, the prototype may be judged inadequate to meet needs and expectations and undergo complete revision, remaining on the left side of the loop.

In the return direction (I to J), X enables feedback from Usage back to Needs, allowing insights from real-world performance and reassessment to inform renewed needs identification and requirements reformulation. Additionally, X allows for alternative governance decisions — such as remaining within the right-hand side of the loop for continued optimisation/maintenance, or redirecting insights from Research directly back into Needs following proof-of-concept failure.

Non-sequential pathways are visualised through dashed lines in the framework and reflect how technologies progress under real-world development conditions. These pathways are enabled by five mechanisms: (EuroScan international network, 2025) the transition point itself; (Banta and Thacker, 1990) flexible entry points, allowing technologies to enter the life cycle at different subprocesses depending on origin, maturity, or prior evidence base; (Jonsson et al., 2002) parallel progression across multiple subprocesses simultaneously; (Greer et al., 2023); non-adjacent subprocess transitions in response to emerging evidence or contextual change; and (Mayer-Foulkes et al., 2021) defined exit points at which technologies may leave the life cycle due to development failure, lack of effectiveness, safety concerns, or disinvestment decisions. Due to this the steps are capsuled by their methodology and can be paralysed by their aim.

### 4.5 Multi-level contextual integration

LC 2.0 explicitly integrates macro-, meso-, and micro-level considerations to capture the context-dependent conditions under which health technologies are developed, implemented, and used.

At the macro level adaptive regulatory approaches such as regulatory sandboxes or conditional approvals may affect Development- and Implementation-related subprocesses (D–G), while reimbursement instruments such as managed entry agreements link coverage decisions to evidence generation in the Usage phase.

At the meso level, organisational readiness, workflow integration, interoperability, leadership support, and workforce capacity determine whether formally approved and reimbursed technologies are successfully adopted in routine practice. Learning from routine use at the organisational level, variation in uptake, outcomes, or resource implications, informs reassessment processes and decisions on adaptation, scaling, or disinvestment.

At the micro level, patient and user involvement shapes early conceptualisation and research activities, while patient-reported outcomes and experiences generated during routine use provide critical insights into perceived benefits, treatment burden, adherence, and acceptability.

### 4.6 Knowledge requirements, stakeholder engagement, and dynamic mechanisms

Within LC 2.0, knowledge is operationalised as contextually situated information that must be fit for purpose and communicated at the right point in the life cycle. Claims of “insufficient knowledge” frequently reflect a mismatch between available evidence and the specific decision context, rather than an absolute absence of data and information. The framework makes knowledge requirements explicit, decision-oriented, and stakeholder-specific: for regulatory authorities, the central question is whether a technology does what it claims and whether it is safe; for HTA bodies and payers, whether it is worth investing and implementing; for healthcare professionals, whether it is clinically meaningful and workable in everyday practice; for patients, whether it improves outcomes that matter in daily life; for industry, whether it can be developed and brought to market responsibly; and for payers, whether it represents value for money and value for the health of the society.

Stakeholder engagement is operationalised by identifying the decision points at which specific questions arise and clarifying which actors are best positioned to contribute. Stakeholders may assume different roles across subprocesses — as formal decision-makers, implementers, or holders of the best available knowledge at a given moment, drawing on scientific evidence, experiential insight, organisational experiences, or system-level information as appropriate.

Dynamic feedback loops constitute a core operational feature of LC 2.0. Knowledge and experience generated during routine use feed back into reassessment, guideline revision, and problem reformulation. Implementation experience informs ongoing development and local adaptation. Patient and user experiences influence both coverage decisions and care pathway refinement. These feedback processes operate at multiple levels, as iterative loops within phases and as structured re-entry pathways across phases, implementing learning health system principles. Continuous quality improvement is embedded in routine use, shifting the Usage phase from a terminal phase to an active learning environment that continuously informs upstream decisions.

### 4.7 Governance architecture

LC 2.0 combines elements from different process paradigms by distinguishing between phase-level governance and subprocess-level flexibility. At the phase level, transitions between Needs, Idea, Research, Development, Implementation, and Usage follow structured governance logics comparable to phase-gate frameworks. Progression between phases is contingent on meeting defined criteria related to evidence adequacy, regulatory compliance, safety requirements, or coverage decisions. These phase transitions represent formal decision points at which accountability, transparency, and justification are required. Within and between phases, however, the framework allows for substantial flexibility at the subprocess level: subprocesses may proceed in parallel, iterative feedback loops between adjacent subprocesses support refinement without full phase transitions, and non-adjacent subprocess movements enable adaptive responses to emerging evidence or contextual change. Dynamic elements are explicitly accommodated at the task level within specific subprocesses, particularly in the Development and Usage phases, where experimentation, optimisation and real-world learning must remain possible while still operating within the formal constraints of regulatory and governance requirements; making this bounded flexibility visible is a core purpose of LC 2.0.

### 4.8 Framework validation and refinement

The framework underwent iterative refinement based on feedback from stakeholders. Feedback highlighted the Idea phase as a particularly important contribution, operationalising learning from real-world performance into renewed needs definition in a way does not present in LC 1.0. Subprocess descriptions were refined to more clearly distinguish development, assessment, implementation, and learning activities. Macro-, meso-, and micro-level influences were more explicitly embedded with examples. Structural and visual elements were also revised: subprocess naming and ordering were updated for clarity; the colour scheme was adjusted for accessibility; and Transition Point X was formally designated as a governance decision and redirection point in the framework structure.

## 5 Discussion

LC 2.0 advances health technology governance by reconceiving the life cycle not primarily as a sequence of regulatory and commercial milestones, but as a structured service for knowledge generation. It is explicitly designed as a cross-silo, interdisciplinary and cross-policy framework. It connects actors and decisions across regulatory, HTA, clinical, public health and industrial policy domains and provides a shared reference for their respective knowledge needs. The central conceptual contribution is the shift from a product life cycle to a knowledge life cycle - a framework that makes explicit what must be learned, by whom, and when, across all phases from needs identification to reassessment. This shift has direct practical implications.

First, it reframes the purpose of post-market activities. Rather than treating real-world use as the end of the life cycle, LC 2.0 positions the Usage phase as an active learning environment from which insights continuously flow back into needs identification, problem reformulation, and renewed innovation. This design aligns with learning health system principles and operationalises the feedback loops that existing frameworks frequently omit (Zych et al., 2024).

Second, the framework enables appropriate technology use by ensuring that evidence is generated and applied at the right point in the life cycle, for the right decision, by the right stakeholders. By making evidence requirements explicit and stakeholder-specific, LC 2.0 addresses the persistent challenge that “insufficient evidence” claims often reflect a mismatch between available knowledge and decision context rather than a genuine absence of data (Goodman, 1998; Wang et al., 2018).

Third, LC 2.0 supports needs-driven innovation through the explicit representation of pull mechanisms in the Needs and Idea phases. The conventional innovation process is predominantly technology-push: products are developed based on technological opportunities and subsequently introduced into healthcare systems (Schurer et al., 2023). LC 2.0 anchors one starting point of innovation in articulated health system needs and policy priorities, providing a governance basis for steering R&D toward areas of genuine societal need. This is particularly relevant for conditions such as antimicrobial resistance and neglected tropical diseases, where market-push dynamics alone have proven insufficient.

Fourth, the framework supports efficient, regulator-appropriate R&D by establishing phase-appropriate evidence standards. Describing the best-available, best-possible and needed evidence is reflecting the different phases and their requirements to reduce or minimise the uncertainty in each phase. Applying evidence requirements such as RCTs to early feasibility decisions is wasteful and may suppress promising innovations. Conversely, permitting low-quality evidence to support regulatory authorisation or coverage decisions places patients and health systems at risk. LC 2.0 operationalises a graduated evidence logic aligned with the 2011 Oxford Centre for Evidence-Based Medicine (OCEBM) Levels of Evidence framework, in which evidence standards increase as decisions become more consequential and accountability requirements rise (Group OLoEW, 2012).

In comparison with existing frameworks, LC 2.0 offers several distinctive contributions. It extends device-focused framework such as the Medical Decide Innovation Consortium (MDIC) framework by systematically covering post-market and implementation phases (Consortium, 2024). It builds on surgical innovation frameworks like IDEAL by integrating implementation science and broader stakeholder engagement (Ergina et al., 2013). It incorporates WHO phase logics while situating them within a broader learning-oriented life cycle. For implementation science, LC 2.0 provides an overarching structure that incorporates contextual determinants analogous to those described by the Consolidated Framework for Implementation Research (CFIR), operationalises progression through implementation phases, and connects real-world outcomes to earlier phases in a manner consistent with RE-AIM logic (Damschroder et al., 2009; Shoup et al., 2015). For HTA specifically, the framework positions assessment not as a culminating event but as one continuous phase within a broader cycle of knowledge generation, encompassing early assessment, managed entry, and reassessment (Xoxi et al., 2022; Esmail et al., 2018; network Ei, 2025). Recent proposals to introduce “life cycle HTA” (LC-HTA) emphasise the systematic sequencing of HTA activities at different points in a technology’s life cycle, particularly in situations where evidence, the technology itself or its clinical context are expected to change over time (Pichler et al., 2024). This focus is conceptually consistent with the 2020 HTA definition, which already describes HTA as determining the value of health technologies at different points in their life cycle, rather than introducing a new paradigm (Pichler et al., 2024). LC 2.0 is compatible with LC-HTA approaches but conceptually broader, as it embeds HTA activities within a multi-stakeholder governance framework that also includes regulatory processes, implementation dynamics and learning-health-system feedback loops; life cycle HTA applications can therefore be seen as specific operational use cases within the LC 2.0 model rather than as an alternative life-cycle concept.

The strengths of this work include its empirical foundation through a multi-method approach, stakeholder inclusivity across six groups, conceptual coherence with LC 1.0, and validated flexibility accommodating heterogeneity across four technology types (Zarghani et al., 2024; Farid, 2019). Use case validation transformed a conceptual proposal into a framework with demonstrated applicability: universal elements, the six phases, needs assessment, real-world evidence, stakeholder engagement, and implementation focus, provide stability, while flexibility mechanisms accommodate legitimate variation in pathways, durations, and emphases (Jaksa et al., 2025; Claire et al., 2024).

LC 2.0 is designed to function as a shared reference framework across different application contexts. Three primary modes of use are envisioned. First, as an analytical mapping instrument. The Framework can be applied to document which phases and subprocesses a specific technology has transversed, which have been skipped or condensed and where evidence requirements are not fully addressed. This application is analogues to how LC 1.0 was employed to situate emerging technologies within a governance context and identify the type and maturity of available evidence. Second, as evidence planning scaffold. LC 2.0 can structure early interaction processes between industry, regulators, HTA agencies and other stakeholders by making explicit which evidence is required at each phase transition and by which stakeholder. Third, as a communication tool across disciplines. The infinity loop structure provides a visually accessible reference that supports alignment between stakeholder who operate within different institutional logics.

The transition from LC 1.0 to LC 2.0 was itself shaped by documented learnings from the original framework’s reception and application. LC 1.0 established the foundational principle that health technologies should be understood as sociotechnical phenomena and that evidence generation is continuous rather than culminating at regulatory approval. In practice, however, users of LC 1.0 reported difficulties in applying it to technologies that entered the life cycle at advanced stages, underwent partial cycles or operated under adaptive regulatory. LC 2.0 addresses this directly through the transition point X, flexible entry and exit mechanisms, and explicit non-sequential pathways. A further learning from LC 1.0 concerns stakeholder granularity. The original framework addressed stakeholders generically, whereas LC 2.0 maps evidence requirements to distinct groups across each phase. Future deployment of LC 2.0 should therefore include stakeholder-specific implementation guides that contextualise the framework for each actor group, and prospective validation studies that test its utility as a planning tool in real-world governance processes.

Limitations should be acknowledged. The contextual specificity of the workshops may limit direct transferability across health systems with substantially different governance structures (Goeree et al., 2011). The four use case types, while selected for diversity, do not encompass all technology categories medical devices, AI-based diagnostic tools, combination products, and organisational innovations warrant further validation. The framework necessarily abstracts and simplifies real-world complexity, with substantial within-phase variation that cannot be fully captured. Use case testing demonstrated the framework’s representational capacity but did not yet measure its impact on governance outcomes, evidence planning, or innovation trajectories (Ahmed et al., 2025; Gomis-Pastor et al., 2024).

Future research priorities include additional use case validation across technology categories not yet examined; prospective studies following technologies in real time to test LC 2.0 as a planning tool; development of technology-specific guidance that maintains the common framework while addressing phase-specific complexities (e.g., iterative software updates in digital health, long-term registry requirements for ATMPs, distributional equity considerations in screening programmes); operationalisation of the evidence framework into practical decision tools; and examination of the framework’s applicability across health systems with different structures, resource levels, and governance traditions.

## 6 Conclusion

The Life Cycle of Health Technologies 2.0 reconceives health technology governance and innovation support as a structured knowledge generation service. By making explicit what must be learned, by whom, and when—across all phases from articulating health needs through post-market reassessment and back to new conceptualisation the framework provides a shared reference framework that supports coordinated evidence generation, appropriate use, needs-driven innovation, and efficient, regulator-appropriate R&D.

Validated across four technology types and refined through structured stakeholder engagement, LC 2.0 offers practical value for all six stakeholder groups: regulatory authorities, HTA agencies, healthcare professionals, patients and patient organisations, industry, and healthcare payers. Its infinity loop architecture, flexible pathway mechanisms, and phase-specific evidence framework make it capable of reflecting real-world innovation trajectories while maintaining the governance rigour required for accountable decision-making.

As health technology governance continues to evolve in response to digital transformation, emerging technology classes, and expanding societal expectations, the LC 2.0 framework, grounded in stakeholder input, empirically validated, and conceptually coherent, provides a foundation for approaches to health innovation that are simultaneously adaptive, equitable, and evidence informed. The core insight it carries forward from LC 1.0 remains essential: health technologies are sociotechnical phenomena, and their governance requires sustained attention to the interactions between technology, actors, and context across the entire life cycle.

## Data Availability

The raw data supporting the conclusions of this article will be made available by the authors, without undue reservation.
