# Supplementary material for: From product to knowledge: the life cycle of health technologies 2.0 as a framework for health system improvement
Source: Front Pharmacol. 2026 Aug 10;17:1869110. doi: 10.3389/fphar.2026.1869110 (PMC13500488; doi:10.3389/fphar.2026.1869110)
Supplement: Supplementary file 1 [file Table1.docx]

Supplementary table 1 (S1)

| **Technology category** | **Use case** | **Type** | **Key issues observed** | **Reference** |
| --- | --- | --- | --- | --- |
| Pharmaceuticals | Biosimilar adoption and implementation in low- and middle income settings | Real-world | Need to align regulatory, reimbursement and procurement processes with value-based principles by capturing societal and stakeholder values in decision support | (1, 2) |
| Digital health technologies | Digital decision‑support tool to stratify pregnant women by risk of early birth | Composite | Data quality and representativeness for algorithm training; risk of bias and unequal performance across subgroups; integration into clinical workflows and responsibility allocation; explaining probabilistic risk estimates to clinicians and patients. | (3-6) |
| Screening programmes | Paediatric screening programme for familial hypercholesterolaemia (FH) as developed and analysed in the EU PERFECTO project, combining universal and cascade screening elements to identify children with FH early and initiate preventive treatment | Real-world | Limited and fragmented programme implementation across countries; variable political and financial commitment; data gaps for long-term and societal outcomes; organisational barriers (follow-up pathways, interoperability) and acceptance issues (awareness among professionals, families) | (7-9) |
| ATMPs | Gene therapy for an inherited haematologic disorder introduced under long‑term outcome‑based contracts | Composite | High upfront costs and budget impact; long‑term outcome uncertainty; need for robust registries and follow‑up; complex contractual arrangements; ethical and equity questions around access and prioritisation. | (10) |

# References

1.	Otte M KZ, Al-Omar HK, Boysen M, Chen Y, Etges APBDS, Gutierrez-Ibarluzea I, Seyam A, Mullen D, Dauben HP. Contextual factors in Value-Based Decision Support to enhance the implementation of biosimilars. In Print. 2025.

2.	Otte M, Dauben HP, Ahn J, Gutierrez Ibarluzea I, Drummond M, Simoens S, et al. Value based healthcare and Health Technology Assessment for emerging market countries: joint efforts to overcome barriers. Expert Rev Pharmacoecon Outcomes Res. 2024;24(9):1061–6.

3.	Panch T, Mattie H, Atun R. Artificial intelligence and algorithmic bias: implications for health systems. Journal of global health. 2019;9(2):020318.

4.	Kim S, Brennan PA, Slavich GM, Hertzberg V, Kelly U, Dunlop AL. Black-white differences in chronic stress exposures to predict preterm birth: interpretable, race/ethnicity-specific machine learning model. BMC Pregnancy Childbirth. 2024;24(1):438.

5.	Obra JK, Singh C, Watkins K, Feng J, Obermeyer Z, Kornblith A. Potential for Algorithmic Bias in Clinical Decision Instrument Development. npj Digital Medicine. 2025;8(1):762.

6.	Zhu Y, Sui D, Wang Z, Hu X, Gu L, Qi Y, et al., editors. Augmenting Clinical Decision-Making with an Interactive and Interpretable AI Copilot: A Real-World User Study with Clinicians in Nephrology and Obstetrics. Proceedings of the 2026 CHI Conference on Human Factors in Computing Systems; 2026.

7.	Otte M G-II, Daccord M, Gaenta M. D2.1 Report on the analysis of existing experiences PERFECTO project, Grant Agreement No. 101083929, . Brussels: PERFECTO – Preventing the PREventable – Familial HyperCholesterolaemia Paediatric Screening for Cardiovascular Health Brussels; 2025 13.01.2025. Report No.: Grant Agreement No. 101083929, Deliverable D2.1.

8.	Otte M CB, Evans E, De Jong T, Geanta M, Anthousi M, Groselj U, Wiegman A, Daccord M, Panayiotou A. D2.3: Report on model appliance to familial hypercholesterolaemia paediatric screening programmes. PERFECTO – Preventing the PREventable – Familial HyperCholesterolaemia Paediatric Screening for Cardiovascular Health; 2025. Report No.: Grant Agreement No. 101083929, Deliverable D2.3.

9.	Otte M CB, Panayiotou A, De Jong T, Gaenta M, Anthousi M, Daccord M. D2.2: Societal ROI model for paediatric screening for familial hypercholesterolaemia. PERFECTO – Preventing the PREventable – Familial HyperCholesterolaemia Paediatric Screening for Cardiovascular Health; 2025. Report No.: Grant Agreement No. 101083929, Deliverable D2.2.

10.	Xoxi E, Rumi F, Kanavos P, Dauben HP, Gutierrez-Ibarluzea I, Wong O, et al. A Proposal for Value-Based Managed Entry Agreements in an Environment of Technological Change and Economic Challenge for Publicly Funded Healthcare Systems. Front Med Technol. 2022;4:888404.
