# Supplementary material for: From product to knowledge: the life cycle of health technologies 2.0 as a framework for health system improvement
Source: Front Pharmacol. 2026 Aug 10;17:1869110. doi: 10.3389/fphar.2026.1869110 (PMC13500488; doi:10.3389/fphar.2026.1869110)
Supplement: Supplementary file 2 [file Table2.docx]

Supplementary Table S2 Literature Search Strategy

| 1. Objective | |
| --- | --- |
| Synthesis aim | Identify and characterise the types of evidence required by different stakeholder groups across health‑technology life‑cycle phases, as a core component of their broader knowledge needs, and describe how evidence hierarchies and quality standards vary by phase and decision context. |
| 2. Databases and sources | |
| Bibliographic databases | PubMed (MEDLINE), Scopus, EMBASE |
| Additional sources | Policy documents and methodological guidance from regulatory authorities (e.g. EMA, FDA), national and international HTA organisations and WHO; reports from professional organisations, patient advocacy groups, payer bodies and industry; reference lists of key retrieved articles (hand‑search). |
| Search periods | Database inception to January 2026 |
| Languages | English, German |
| Search concepts and term sets | |
| Concept 1 – Technology domain | health technolog*; medical device*; pharmaceutical*; drug*; digital health; eHealth; mHealth; screening program*; advanced therapy; ATMP; biologics; gene therapy; cell therapy |
| Concept 2 – Life-cycle phase | life cycle; lifecycle; health technology life cycle; development; pre‑market; regulatory approval; market authorisation; implementation; adoption; coverage; reimbursement; HTA; post‑market; real‑world use; reassessment; disinvestment; horizon scanning; early awareness |
| Concept 3 – Evidence requirements | evidence requirement*; evidentiary requirement*; evidence need*; evidence standard*; data requirement*; evidence hierarchy; evidence level*; real‑world evidence; RWE; clinical effectiveness; comparative effectiveness; cost‑effectiveness; patient‑reported outcome*; PRO; implementation outcome*; evidence generation |
| Concept 4 – Stakeholder context | stakeholder*; regulatory authorit*; health technology assessment; HTA; payer*; insurer*; commissioner*; healthcare professional*; clinician*; patient*; industry; manufacturer*; investor*; health system governance; decision‑making; evidence‑based |
| Database search strategy | In each database, Concepts 1–4 were combined using Boolean AND, with appropriate field tags (e.g. title/abstract in PubMed, TITLE‑ABS‑KEY in Scopus, ab,ti and indexing terms in EMBASE). Full search strings are available from the authors on request. |
| 4. Inclusion and exclusion criteria | |
| Inclusion criteria | (1) Addresses health technologies; (2) Explicitly describes evidence requirements, evidence expectations or evidentiary alignment for at least one stakeholder group (regulatory authorities, HTA agencies, payers, clinicians, patients, industry, investors, or cross‑sectoral policy representatives); (3) Links evidence to life‑cycle phases, decision stages or governance questions (e.g. market entry, coverage, implementation, reassessment, disinvestment); (4) Conceptual or empirical study, policy document, methodological guidance or position paper. |
| Exclusion criteria | (1) Technical clinical‑trial reports or pre‑clinical studies without discussion of decision‑making, governance or evidence requirements; (2) Commentaries/editorials without description of evidence needs; (3) Topics unrelated to health technologies or health‑system governance; (4) Non‑English or non‑German publications in database searches. |
| Screening and selection | Titles and abstracts (and, where necessary, full texts) screened against inclusion/exclusion criteria by one reviewer; borderline cases discussed among all authors. Reference lists of included articles and key HTA/regulatory guidance documents were hand‑searched to identify additional records. Further policy and methodological documents from major agencies and organisations were purposively added. |
| Data extraction and synthesis | For each source, we extracted: (a) stakeholder group(s) addressed; (b) evidence types described (e.g. clinical effectiveness, safety, economic, implementation, RWE, PROs, qualitative); (c) life‑cycle phase or decision context (e.g. pre‑market, regulatory approval, coverage, post‑market, reassessment); (d) explicit statements on evidentiary standards, hierarchies or alignment needs. Extracted statements were coded thematically by stakeholder group and life‑cycle phase, and recurring evidence types and quality standards were mapped to the eight stakeholder groups to construct the stakeholder–evidence matrix. |
